# A machine learning-driven web application for sign language learning

**DOI:** 10.3389/frai.2024.1297347

**Published:** 2024-06-18

**Authors:** Hope Orovwode, Oduntan Ibukun, John Amanesi Abubakar

**Affiliations:** ^1^Department of Electrical and Information Engineering, Covenant University, Ota, Nigeria; ^2^Department of Computer Science and Engineering, University of Bologna, Bologna, Italy

**Keywords:** machine learning, sign language recognition, CNN, sign language, American Sign Language (ASL), Python

## Abstract

Addressing the increasing demand for accessible sign language learning tools, this paper introduces an innovative Machine Learning-Driven Web Application dedicated to Sign Language Learning. This web application represents a significant advancement in sign language education. Unlike traditional approaches, the application’s unique methodology involves assigning users different words to spell. Users are tasked with signing each letter of the word, earning a point upon correctly signing the entire word. The paper delves into the development, features, and the machine learning framework underlying the application. Developed using HTML, CSS, JavaScript, and Flask, the web application seamlessly accesses the user’s webcam for a live video feed, displaying the model’s predictions on-screen to facilitate interactive practice sessions. The primary aim is to provide a learning platform for those who are not familiar with sign language, offering them the opportunity to acquire this essential skill and fostering inclusivity in the digital age.

## 1 Introduction

In today’s diverse and interconnected world, fostering effective communication is of paramount importance. A significant portion of the global population faces hearing impairment, which can greatly hinder their ability to engage in meaningful verbal conversations. According to the World Health Organization (WHO), approximately 6% of the world’s population experiences some form of hearing impairment, with nearly 5% requiring therapeutic interventions for “disabling” hearing loss (World Health Organization, 2021; Madhiarasan, 2022; Rodríguez-Moreno et al., 2022). Alarmingly, projections indicate that by the year 2050, as many as 900 million individuals, or one in every ten people, could be affected by debilitating hearing loss (Elsayed and Fathy, 2020; Altememe, 2021).

The impact of hearing impairment extends far beyond the auditory realm, affecting a person’s ability to connect with others, access education, find employment, and participate fully in society (Lee et al., 2022; Al-Hassan et al., 2023). In this context, sign language has emerged as a crucial mode of communication for the Deaf and hard-of-hearing community (Sruthi and Lijiya, 2019; Wadhawan and Kumar, 2020; Ganguly and Deb, 2022). Sign languages, which vary across regions and cultures (Rastgoo et al., 2021; Kındıroglu et al., 2023), provide a visual and gestural means of expressing thoughts and ideas (Thakur et al., 2020; Kodandaram et al., 2021). They offer a bridge to the world for those who may not have access to spoken language. However, in some regions, such as Nigeria, there is no distinctive Nigerian Sign Language (NSL) established for widespread use, despite the existence of over 400 spoken languages and numerous dialects. Consequently, American Sign Language (ASL) is more commonly used in schools for the deaf in Nigeria, while NSL is still in development.

However, learning sign language can be challenging, and access to quality educational resources is often limited (Areeb et al., 2022). Also, acquiring adequately trained translators in any of the numerous signed languages often requires much time and resources (Sharma and Kumar, 2021) and may also hinder the privacy of the hearing-impaired person (James et al., 2022).

In recent years, advances in technology have led to the development of various systems aimed at bridging the communication gap for the deaf and hard-of-hearing community. One notable area of progress is sign language recognition, a field that has garnered significant attention due to its potential to enhance accessibility and inclusion for a marginalized group of individuals. Sign Language Recognition (SLR) has greatly benefited from the integration of machine learning techniques, which have significantly improved the accuracy and efficiency of sign language interpretation by enabling algorithms to adapt and learn from data, leading to more effective communication for individuals with hearing impairments (Abdulkareem et al., 2019; Alausa et al., 2023). Sign Language Recognition (SLR) endeavors to create algorithms and techniques capable of accurately discerning a series of expressed signs and conveying their meanings in the form of written text or spoken language (Kamal et al., 2019).

In our pursuit of this noble goal, we embarked on a journey that commenced with the presentation of our initial findings in a conference paper (Orovwode et al., 2023). In that paper, we introduced a robust sign language recognition system, which represented a significant step forward in sign language technology. We showcased promising results in terms of accuracy and usability, laying the groundwork for future exploration.

Building upon this foundation, this journal article serves as a comprehensive expansion of our research endeavors. While our conference paper offered a glimpse into the potential of sign language recognition, this article delves deeper into the evolution of our system, with a particular emphasis on the novel aspect of deploying the application on the web. We recognized that true progress in accessibility lies not only in the efficacy of our recognition algorithms but also in the ease of access and usability of the technology for a broader audience.

The motivation for our extended research stems from the understanding that, while technology has the potential to break down communication barriers, its impact remains limited if it is not readily accessible to those who need it most. The web, as a ubiquitous platform, offers a unique opportunity to democratize sign language recognition technology, allowing individuals with hearing impairments, educators, caregivers, and the public to access and utilize this valuable tool (Ginsburg, 2020). Therefore, this article chronicles our efforts to adapt and deploy our sign language recognition system on the web, ultimately enhancing its accessibility and usability.

The aim of this paper is to present the development of a web application tailored for the acquisition of the basic American sign language alphabet. The user activates their camera to make hand movements or signs and is shown a word to spell. If the system detects that all the letters of the word have the signed correctly, it gives the user a point. Harnessing the capabilities of machine learning, this application aims not only to expedite the learning process but also to foster inclusivity and bridge the communication gap between the hearing and deaf communities.

The subsequent section of this paper will discuss some related works on the development of sign language recognition systems.

## 2 Review of related works

Katoch et al. (2022) introduced a technique that employs the Bag of Visual Words model (BOVW) to effectively recognize Indian sign language alphabets and digits in real-time video streams, providing both text and speech-based output. The authors utilized skin color-based segmentation and background subtraction for segmentation. By utilizing SURF features and generating histograms to map signs to corresponding labels, the system employed Support Vector Machine (SVM) and Convolutional Neural Networks (CNN) for classification. Additionally, the paper emphasizes user-friendliness through the development of an interactive Graphical User Interface (GUI).

Baktash et al. (2022) presents a visual-based translator for converting sign language into speech. It employs a hand gesture classification model with Region of Interest (ROI) identification and hand segmentation using a mask Region-based Convolutional Neural Network (R-CNN). The model, trained on a substantial dataset using Convolutional Neural Network (CNN) deep learning, achieved a high accuracy of 99.79% and a low loss of 0.0096. The trained model is hosted on a web server and loaded onto an internet browser using a specialized JavaScript library. Users capture hand gestures with a smart device camera for real-time predictions, addressing communication challenges for individuals unable to speak.

Kasapbaşi et al. (2022) in their paper, contributed to the field of sign language recognition (SLR) through the development of an American sign language recognition dataset and the implementation of a Convolutional Neural Network (CNN) model. By utilizing neural networks, the study focused on interpreting gestures and hand poses of sign language into natural language. The dataset introduced in the research is characterized by its consideration of various conditions such as lighting and distance, setting it apart as a novel addition to the SLR domain. The model demonstrated an accuracy of 99.38% across diverse datasets.

The study conducted by Bendarkar et al. (2021) proposed a comprehensive design and architecture for American Sign Language (ASL) recognition using convolutional neural networks (CNNs). The approach incorporates a pre-trained VGG-16 architecture for static gesture recognition and a complex deep learning architecture featuring a bidirectional Convolutional Long Short Term Memory network (ConvLSTM) and a 3D convolutional neural network (3DCNN) for dynamic gesture recognition. This innovative architecture aims to extract 2D spatiotemporal features. Accuracy tested against hand gestures for ASL letters captured by webcam in real time was determined to be 90%.

The authors (Yirtici and Yurtkan, 2022) proposed a novel method for recognizing characters in Turkish Sign Language (TSL) using a transfer learning approach with a pre-trained Alexnet and a Region-based Convolutional Neural Network (R-CNN) object detector. The method achieved a commendable 99.7% average precision in recognizing TSL signs, demonstrating its efficacy compared to traditional methods. This study is notable for successfully applying transfer learning to TSL and opens avenues for further enhancement in sign image representations.

Das et al. (2023) presented a study on the automatic recognition of Bangla Sign Language (BSL) using a hybrid model comprising a deep transfer learning-based convolutional neural network and a random forest classifier. The proposed model was evaluated on ‘Ishara-Bochon’ and ‘Ishara-Lipi’ datasets, which are multipurpose open-access collections of isolated numerals and alphabets in BSL. With the incorporation of a background elimination algorithm, the system achieved notable accuracy, precision, recall, and f1-score values: 91.67, 93.64, 91.67, 91.47% for character recognition, and 97.33, 97.89, 97.33, 97.37% for digit recognition, respectively.

The authors (Soliman et al., 2023) introduce a recognition system for Argentinian Sign Language (LSA) that leverages hand landmarks extracted from videos within the LSA64 dataset to differentiate various signs. The hand landmarks’ values undergo transformation via the Common Spatial Patterns (CSP) algorithm—a dimensionality reduction technique with a history in EEG systems. Extracted features from the transformed signals are then employed as inputs for diverse classifiers like Random Forest (RF), K-Nearest Neighbors (KNN), and Multilayer Perceptron (MLP). Through a series of experiments, the paper reports accuracy rates ranging from 0.90 to 0.95 for a collection of 42 distinct signs.

Lu et al. (2023) presented a novel wearable smart glove for gesture language communication, addressing limitations in existing solutions for the hearing-impaired. By integrating strain-sensor arrays and machine learning, the glove achieved over 99% accuracy in recognizing gestures, offering real-time feedback and applications across industries like entertainment, healthcare, and sports training. The strain sensors employed SF-hydrogel as the sensing layer, capitalizing on its flexible nature, strong biocompatibility, easy fabrication process, and impressive conductive properties.

Eunice et al. (2023) introduced a systematic approach to address the challenges of gloss prediction in word-level sign language recognition (WSLR) by leveraging the Sign2Pose Gloss prediction transformer model. The proposed method adopted hand-crafted features over automated extraction for improved efficiency and accuracy. Notably, a refined key frame extraction technique using histogram difference and Euclidean distance metrics was introduced to select essential frames. Augmentation techniques and pose vector manipulation were employed to enhance model generalization. YOLOv3 was incorporated for normalization, aiding in signing space detection and hand gesture tracking. Experimental results on WLASL datasets exhibited remarkable achievements, with a top 1% recognition accuracy of 80.9% in WLASL100 and 64.21% in WLASL300, surpassing state-of-the-art approaches. The amalgamation of key frame extraction, augmentation, and pose estimation notably boosted the model’s performance by enhancing precision in detecting subtle variations in body posture, leading to a significant 17% improvement in the WLASL 100 dataset.

The paper (Kumar, 2022) addressed the challenges in recognizing British Sign Language (BSL) fingerspelling alphabet using a Deep learning framework. By employing Convolutional Neural Network (CNN), the work improved upon traditional methods by achieving higher precision, recall, and F-measure percentages. The model’s performance surpassed previous approaches on BSL corpus dataset and webcam videos, reporting a notable accuracy of 98.0% for a broad lexicon of words.

An overview of the research studies that have been discussed can be found in Table 1.

**Table 1:** Overview of the mentioned studies.

Data collection method	Classification method	Dataset
Video frames	Bag of Visual Words model (BOVW)	Indian Sign Language (ISL) dataset alphabet + digits (0–9)
Captured hand gestures with a smart device camera	Convolutional Neural Network (CNN)	American Sign Language
Static images	Convolutional Neural Network (CNN)	American SL alphabet dataset
Static images and videos	Pretrained VGG-16 architecture for static gesture recognition, bidirectional convolutional Long Short Term Memory network (ConvLSTM) and 3D convolutional neural network (3DCNN) for dynamic gesture recognition.	American SL alphabet dataset
Captured images containing signs, extracted from video files	Alexnet and Region-based Convolutional Neural Network (R-CNN) object detector	Turkish SL dataset
Static images	Convolutional neural network and a random forest classifier	Bangla Sign Language: ‘Ishara-Bochon’ and ‘Ishara-Lipi’ datasets.
Hand landmarks extracted from videos	Random Forest (RF), K-Nearest Neighbors (KNN), and Multilayer Perceptron (MLP).	LSA64 dataset
Static images	Strain-sensor arrays and machine learning models	Chinese Sign Language dataset
Video frames	Sign2Pose Gloss prediction transformer model	WLASL datasets
Video frames	Convolutional Neural Network	BSL corpus dataset

A noticeable gap in the existing literature is the integration of sign language recognition into web-based applications. Prior research has primarily focused on offline or specialized settings, leaving the web application aspect unexplored. This distinct focus is the cornerstone of our work, as we extend our sign language recognition system to the web, addressing a critical need that previous studies have not fully embraced. This expansion not only builds upon prior foundations but also pioneers the accessibility and usability of sign language recognition technology for a broader audience (see Table 2).

**Table 2:** Comparison of results of model simulation against previous literature.

Author	Algorithm	Accuracy
Baktash et al. (2022)	Convolutional Neural Network (CNN)	99.79%
Kasapbaşi et al. (2022)	Convolutional Neural Network (CNN)	99.38%
Bendarkar et al. (2021)	VGG-16, 3D-CNN	97.50%
Kumar (2022)	Convolutional Neural Network (CNN)	98.0%
Yirtici and Yurtkan (2022)	AlexNet	99.7%
Orovwode et al. (2023)	Convolutional Neural Network (CNN)	94.68%

## 3 Methodology

In this section, we delve into the methodology employed to realize the seamless deployment of our sign language recognition system model on the web. Building upon the foundation laid in our previous work (Orovwode et al., 2023), we explore the technical adaptations and innovations necessary to transition our system from its original offline setting to the dynamic and interactive environment of the web.

### 3.1 Sign language recognition system model (brief recap)

In alignment with the methodology outlined in our previous paper (Orovwode et al., 2023), the development and deployment of our web-based sign language recognition system encompassed several crucial stages, which are summarize below:

#### 3.1.1 Data acquisition

**Figure 1:**
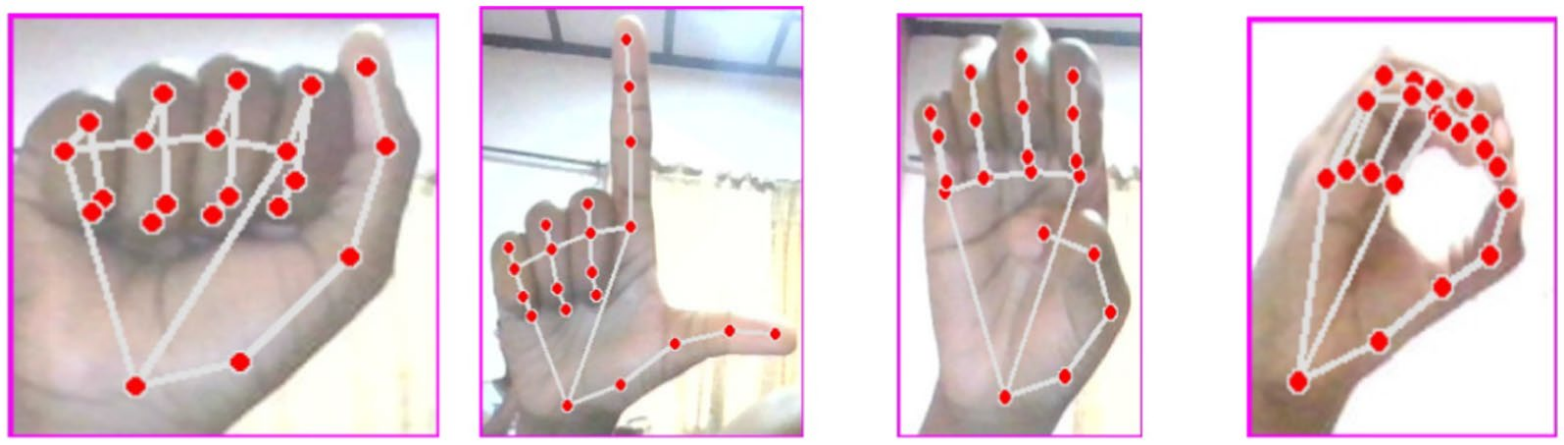
Sample of the dataset (Orovwode et al., 2023).

The acquisition of our image dataset involved the use of a laptop camera to capture the images with the help of the Python OpenCV library. The CVzone HandDetector module aided in detecting the signer’s hand in the camera’s field of view. After detection, the hand signs were cropped and resized to a consistent 300 × 300-pixel size using OpenCV. Our dataset consisted of 44,654 images saved as JPG files, with 24 image classes after excluding dynamic signs J and Z. We divided the dataset into a training set (70%) and a test set (30%) for model training and validation, resulting in 14,979 instances in the test set. Sample of the dataset is seen in Figure 1.

#### 3.1.2 Data pre-processing

The pre-processing stage involved image resizing to 224×224 pixels to suit the model’s requirements and normalization of pixel intensity values. This normalization ensured that all input data shared a similar scale. The datasets were transformed into one-hot encoded vectors using the ‘to_categorical’ function from the Keras library. Additionally, pixel intensity values were adjusted to achieve a mean of 0 and a variance of 1, enhancing computation speed via hardware acceleration.

#### 3.1.3 Model training

We developed our model using the Keras framework, employing a convolutional neural network (CNN) architecture. The model consisted of three convolutional layers with ReLU activation and a max-pooling layer. The number of filters in these layers progressively increased (32, 64, 128). Convolutional outputs were flattened and passed through fully connected layers with ReLU activation for image classification. The output layer, featuring Softmax activation, comprised 24 neurons representing the 24 sign language classes. Model training was conducted for 5 epochs to mitigate overfitting, considering the dataset’s limited size.

#### 3.1.4 Model evaluation

We utilized the test dataset to assess our model’s performance on unseen data. We computed various metrics, including accuracy, precision, recall, and F1-score, to evaluate the model’s effectiveness in sign language recognition. The values obtained are reported in our previous paper. Upon evaluation, the model demonstrated an accuracy level of 94.68%.

### 3.2 Model deployment

The machine learning model was integrated into a web application to provide an intuitive interface for users to interact with the system. The web application was built using HTML, CSS, and JavaScript for the front-end, and Flask for the back-end. The system’s user interface was created to be easy-to-use and accessible to all users, with a straightforward and intuitive design. Users can interact with the system through their webcam, and the interface provides clear instructions and feedback on the screen. The application was set up to receive input data from the user’s webcam and feed it into the model for real-time prediction of sign language gestures. The output was then displayed on the user’s screen in the form of text and a point is given to the user for each word spelled correctly.

#### 3.2.1 Expanded explanation of system integration

1 Front-end implementation:

- HTML was used to structure the web pages, while CSS is used to style these elements, ensuring a user-friendly interface.
- JavaScript (AJAX): JavaScript, particularly through AJAX, facilitates real-time communication between the client-side and server-side. When a user submits a hand gesture, an AJAX request is sent to the Flask server with the image data.

2 Back-end processing with flask:

- Flask acts as the server-side framework, handling incoming requests and interfacing with the CNN model to process the hand gesture images. The endpoint /predict receives the image data, preprocesses it, and feeds it into the trained CNN model to predict the corresponding ASL letter.

3 CNN model integration:

- The CNN model, implemented in a Python environment, is loaded into the Flask application at runtime. This model processes the input images and returns the predicted ASL letter.

4 Scoring system:

- Each time a user spells a word correctly, the system verifies the sequence of predicted letters against the target word. If all letters match, the user earns a point.
- The scoring logic is implemented on the client side, updating the user’s score in real-time based on the server’s predictions.

5 Development environment:

- The development environment for our web application includes Flask, TensorFlow/Keras for the CNN model, and typical front-end technologies (HTML, CSS, JavaScript).
- We used a virtual environment for Python dependencies management, ensuring that all required libraries (Flask, TensorFlow, etc.) are correctly installed.

The sign language web app follows a specific sequence to generate predictions. Here’s how the process unfolds:

1. User starts by accessing the home page of the app. They are presented with a choice between two modes: easy and hard.
2. Regardless of the mode chosen, the app will utilize the user’s webcam. Users will use their hands to input responses, effectively spelling out words in sign language.
3. If the user selects the easy mode, the screen will display images of hand signs that the user can mimic to spell the given word, shown in white text. However, the screen will not show any hand sign images in the hard mode.
4. The program then processes the user’s input data. It detects the signs made by the user to spell out the word. If the user gets any letters correct, those letters are highlighted in green.
5. Users can continue practicing their word spelling in their chosen mode until they develop confidence in their ability to communicate through sign language accurately.

**Figure 2:**
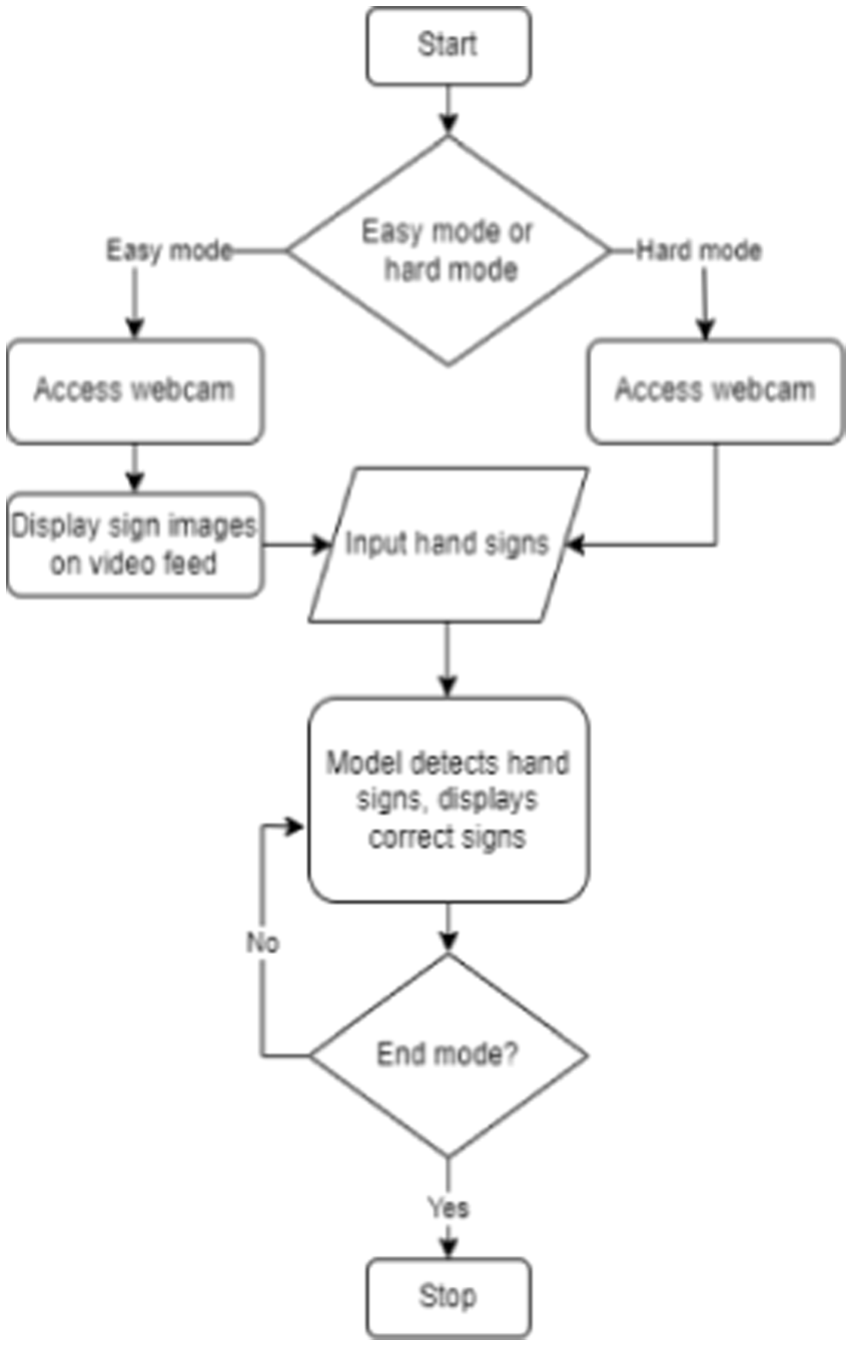
Web application flowchart.

**Figure 3:**
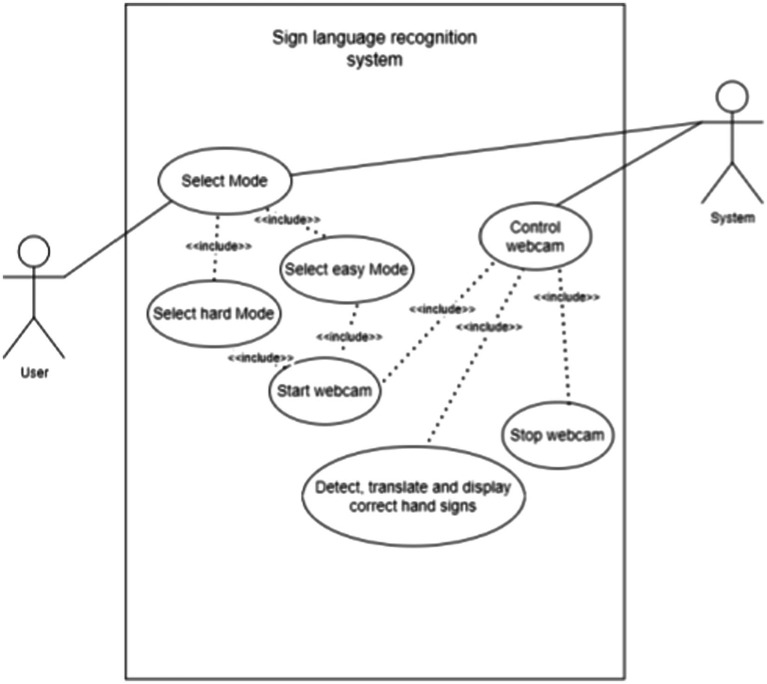
Use case diagram.

The flowchart of the process is shown in Figure 2. Figure 3 shows the use case diagram of the application, which illustrates how the components of the application interact.

### 3.3 Hardware requirements

Below is a detailed list of the hardware components required to run this application.

1. Processor (CPU): 11th Gen Intel(R) Core(TM) i5-1135G7 @ 2.40GHz, 2,419 Mhz, 4 Core(s), 8 Logical Processor(s)
2. Memory (RAM): 8GB to 16GB RAM
3. Graphics Card: Intel(R) Iris(R) Xe Graphics
4. Network Requirements: Must have a strong internet connection.

## 4 Results and discussion

In this section, we present the outcomes of our efforts to build and deploy the web-based sign language learning application, leveraging the methodologies established in our previous work.

Our simulated model, although slightly lower, still attains a respectable accuracy level based on the comparison of results to those in previous literature. We deployed the model onto the web application based on the favourable results obtained from our model.

The model, once deployed, successfully identified sign language alphabet gestures and proficiently converted them into their corresponding text forms, maintaining a high level of precision. The source code for the application can be found at https://github.com/ibukunOduntan/SignLangApp. Additionally, the application can be accessed at as-learn.com.

**Figure 4:**
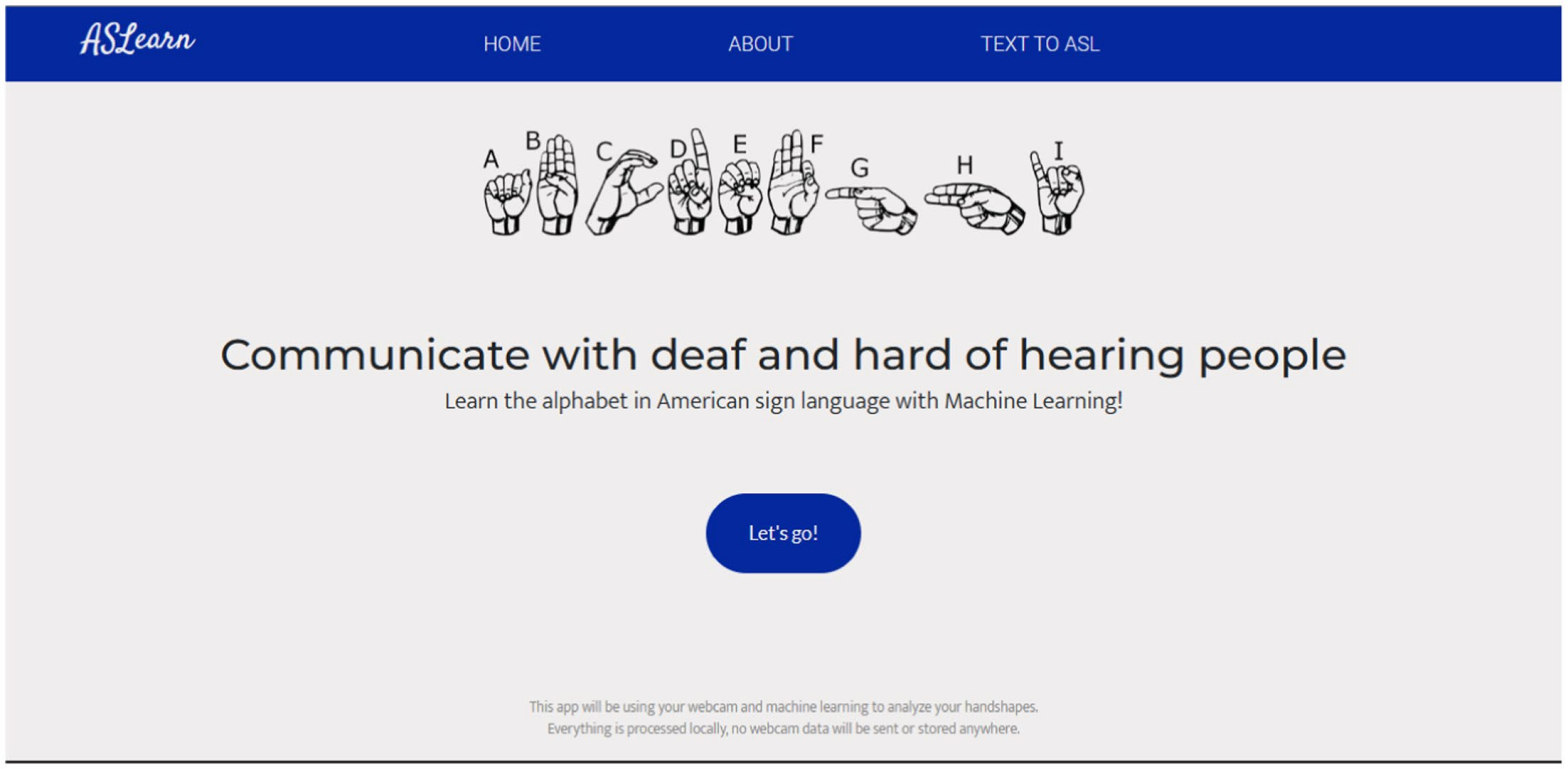
Landing page of the application.

**Figure 5:**
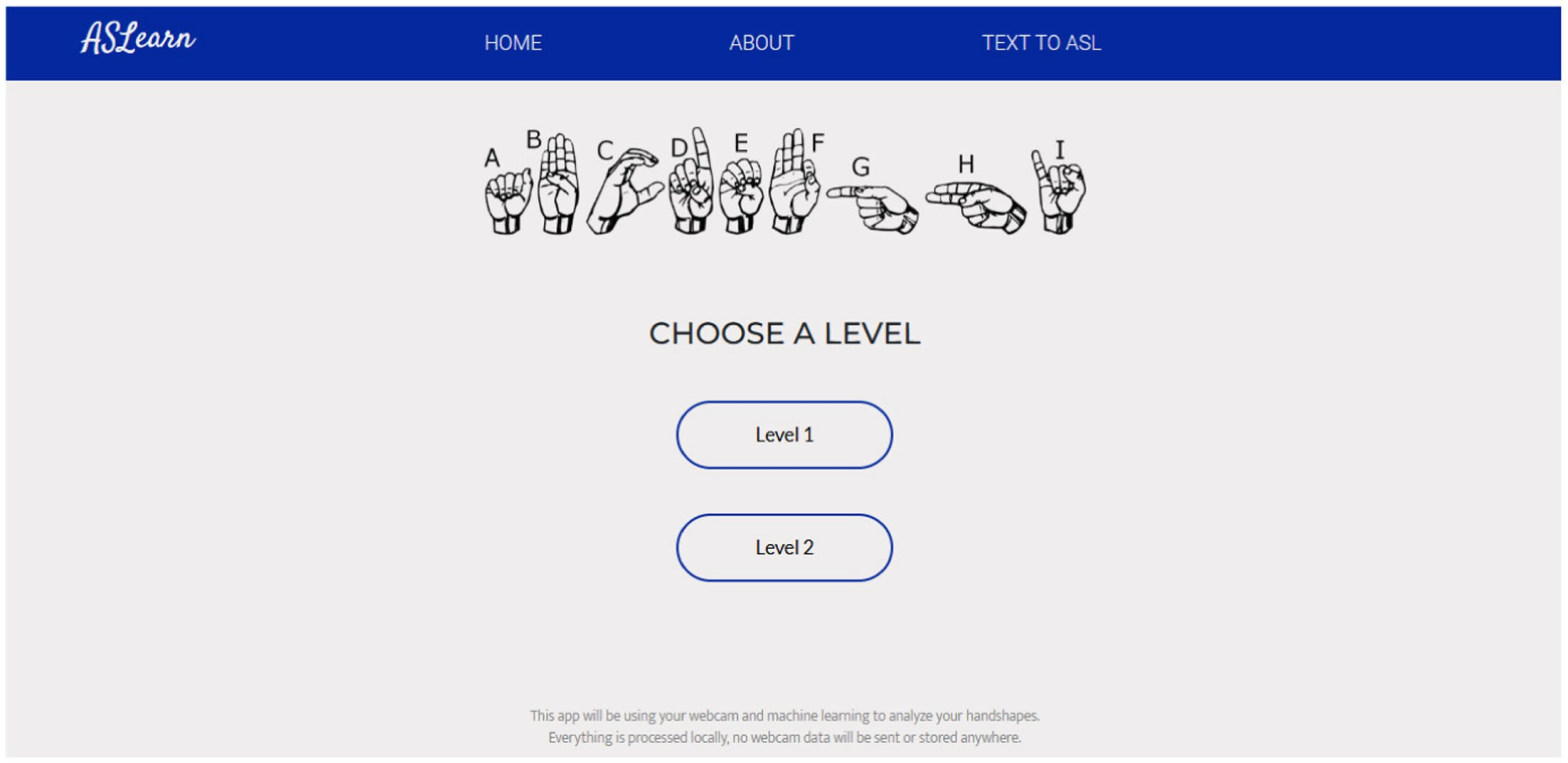
Page where the user selects a level.

Refer to Figure 4 for a depiction of the web application’s homepage. Additionally, Figure 5 showcases the page where users select their preferred difficulty level for engagement.

**Figure 6:**
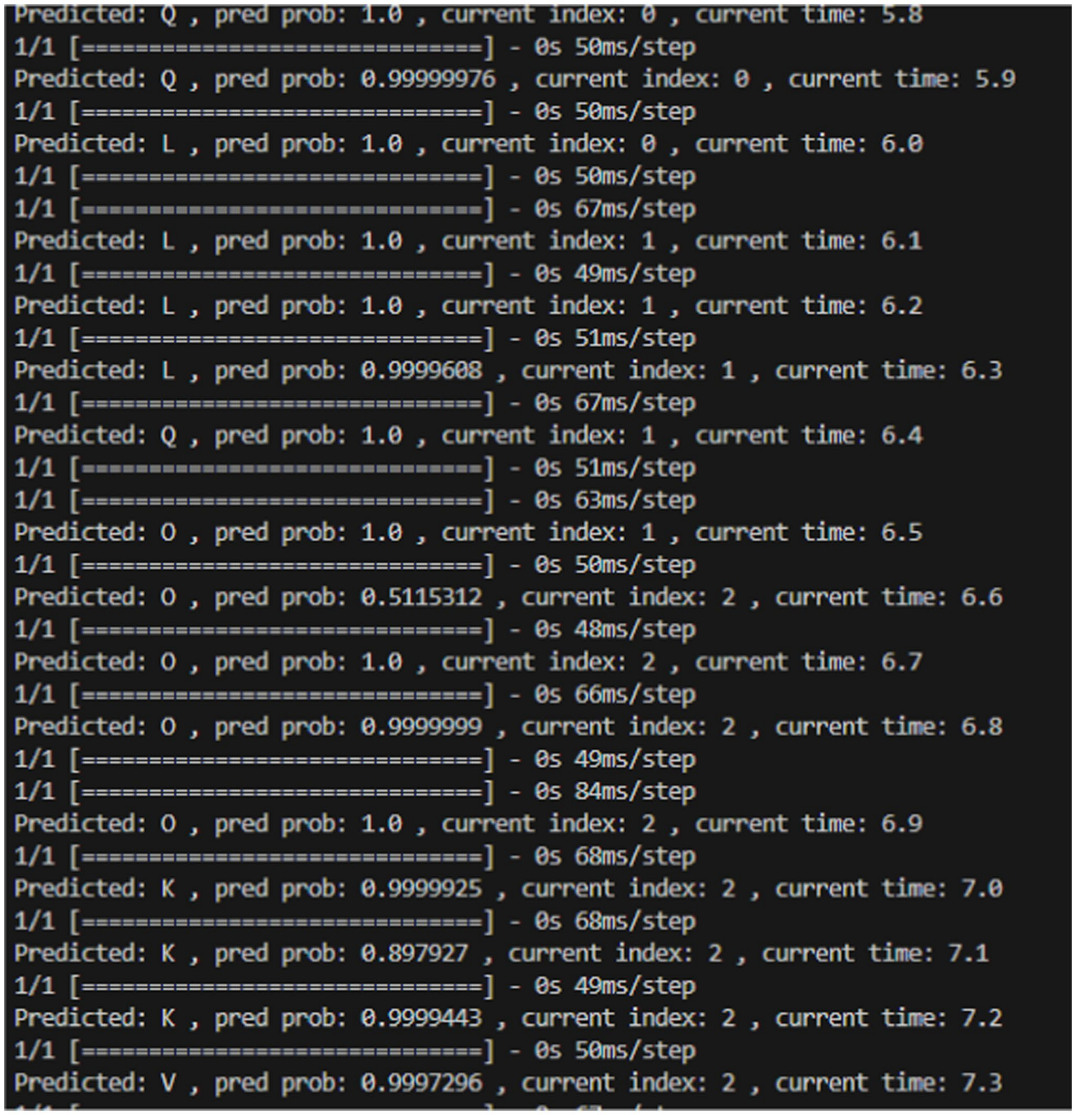
Series of output showing prediction probability and latency of prediction at each time step.

The average latency per step was calculated to be 64.02 ms/step. Latency indicates the time it takes for the model to make predictions at different time points. Minimizing latency is crucial for ensuring that the system can respond to users’ gestures promptly, contributing to a more responsive and user-friendly experience. The low average latency is indicative of an efficient system that can promptly respond to user input. Figure 6 depicts a brief series of outputs that show the prediction probability and the latency of prediction at each time step.

### 4.1 User feedback

To assess the usability and effectiveness of the sign language learning web application, a structured feedback mechanism was employed. A Google Form was created, soliciting user feedback on various aspects of the application’s performance. Specifically, users were asked to rate the application on a scale from 1 to 5 in the following areas: ease of use, accuracy and performance of the Convolutional Neural Network (CNN) model, latency of the application, and learning effectiveness. Additionally, users were encouraged to provide any additional feedback or suggestions for improvement.

**Figure 7:**
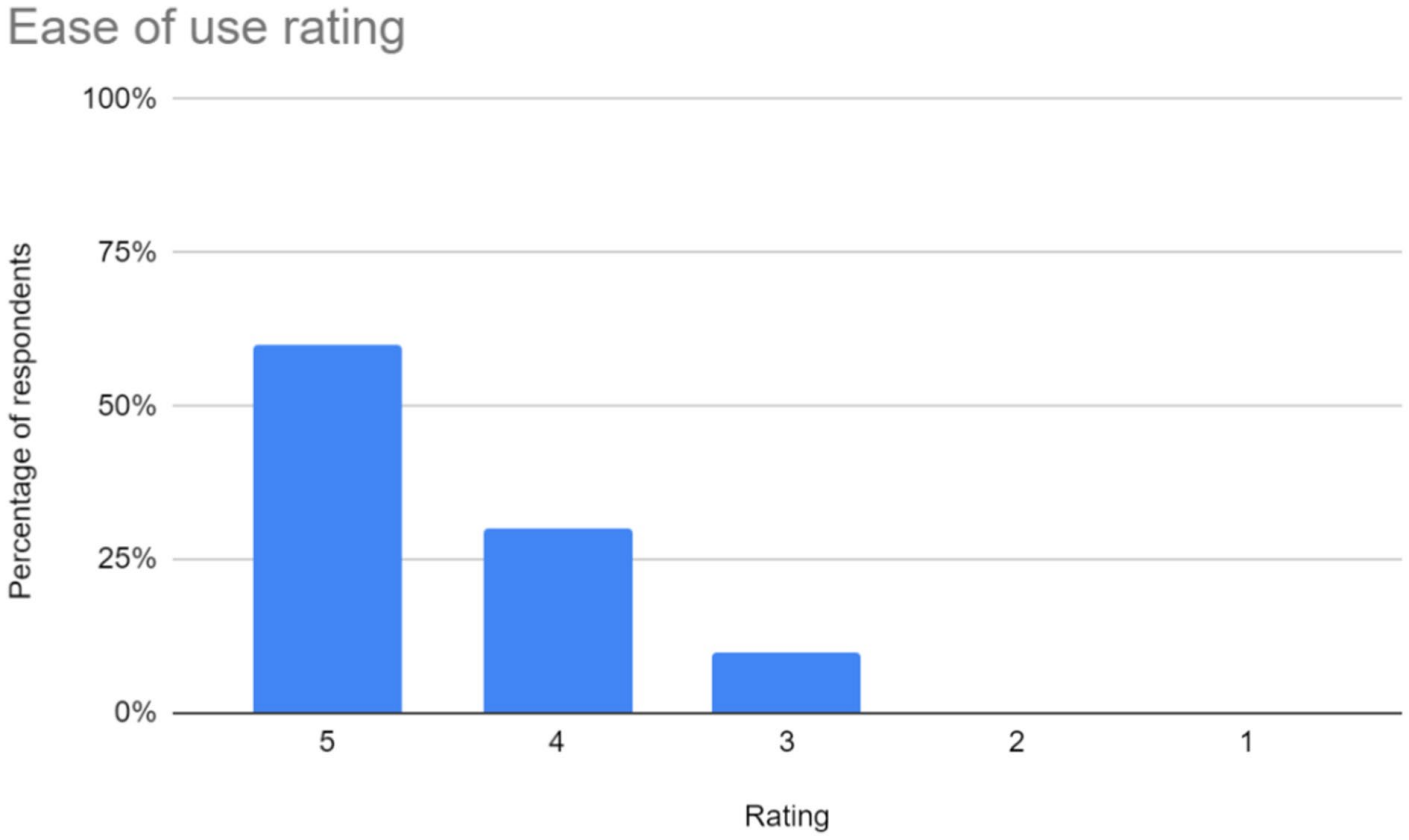
Ease of use ratings for the sign language learning application. The chart in Figure 6 shows that 60% of users rated the application as very easy to use (score of 5), while 30% rated it as easy to use (score of 4). However, 10% found it somewhat less easy (score of 3). Overall, the majority of users found the application to be user-friendly.

**Figure 8:**
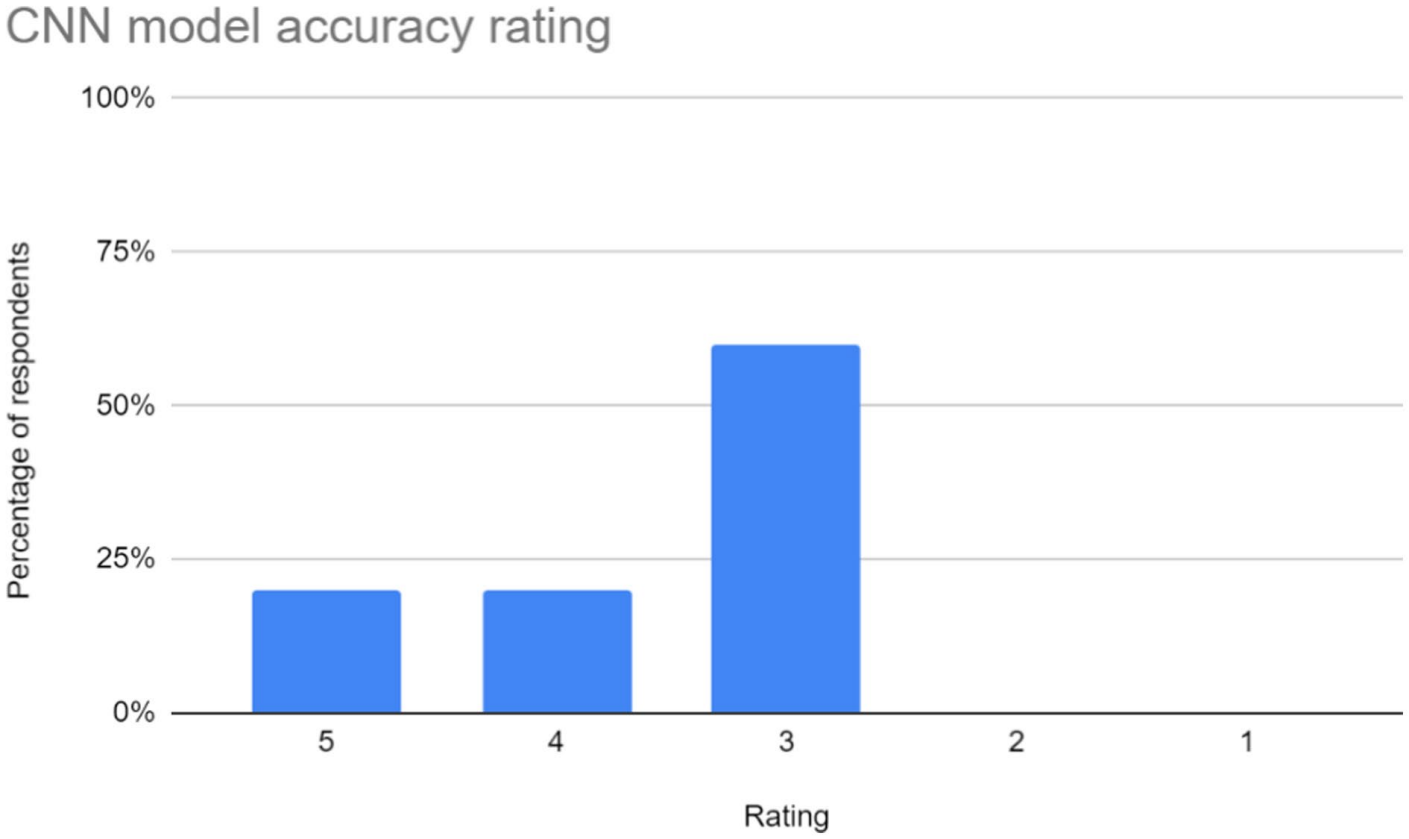
CNN model accuracy ratings for the sign language learning application. The distribution of ratings shows that 20% of respondents rated the CNN model's accuracy as 5, another 20% rated it as 4, and the majority, 60%, rated it as 3. This suggests that there may be room for improvement in terms of consistency or overall performance. Overall, these ratings imply that while some users found the model to be accurate in recognizing sign language gestures, others may have experienced issues or perceived limitations.

**Figure 9:**
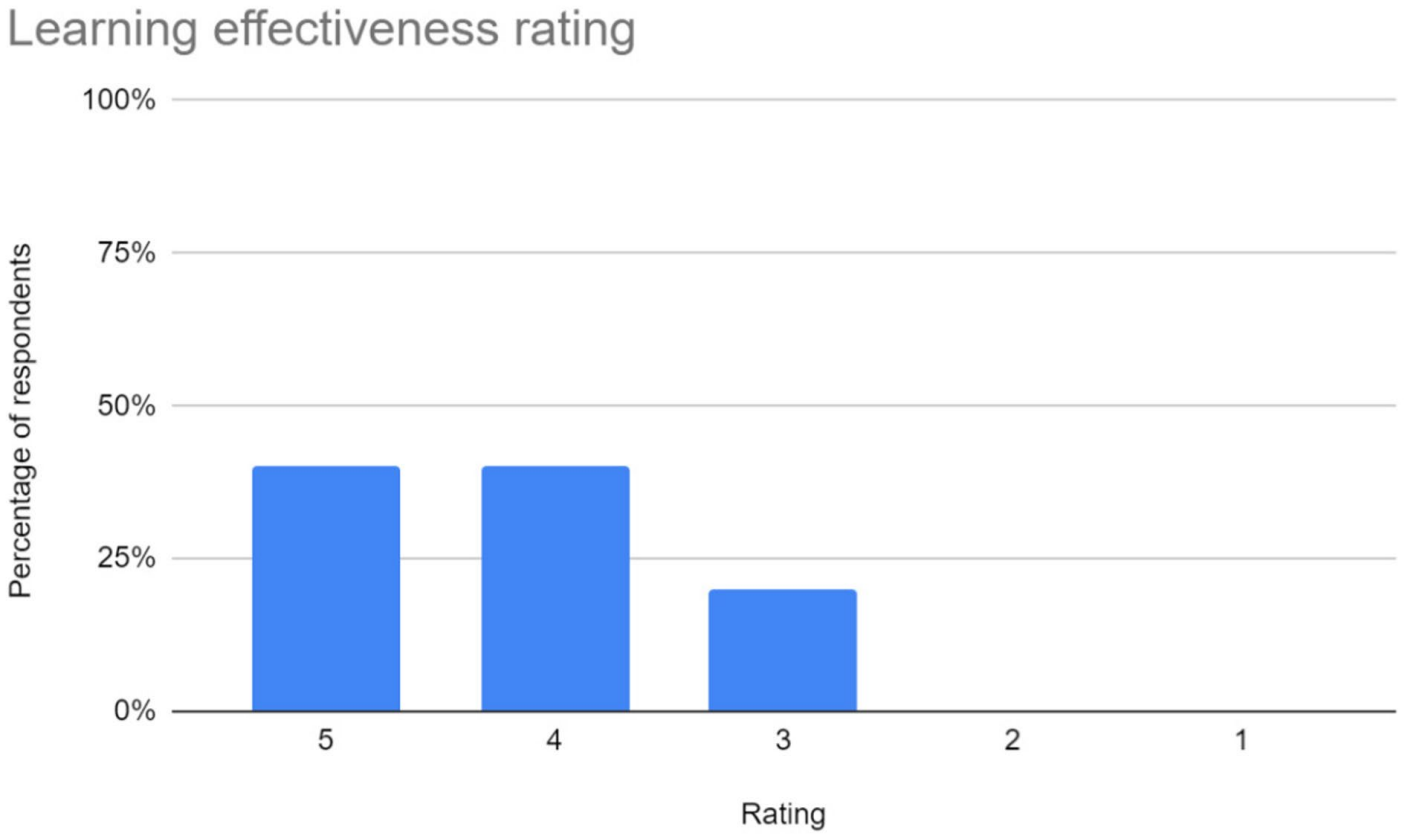
Learning effectiveness ratings for the sign language learning application.

The feedback gathered through the Google Form was analyzed to gain insights into user perceptions and experiences with the application. Barcharts were utilized to visually represent the summarized responses, providing a clear overview of user sentiments across different evaluation criteria. The following subsections provide a detailed analysis of the feedback received in each area:

1. Ease of use: users rated the application based on its navigational simplicity and user-friendly interface. The chart representing user ratings in this aspect is shown in Figure 7.
2. Accuracy and Performance of the CNN Model: The effectiveness of the CNN model in interpreting and recognizing sign language gestures was evaluated by users. The bar chart in Figure 8. depicts user ratings in terms of accuracy and performance.
3. Latency of the application: users assessed the responsiveness and speed of the application in interpreting gestures and providing feedback. “A majority of users reported experiencing latency ranging from mild to severe during their interaction with the application. The reported delays may be attributed to the model’s struggle to generalize across different hand orientations or shapes, possibly due to overfitting to specific patterns observed during training. To address this, expanding the dataset to include a wider variety of signers and hand orientations could help improve the model’s ability to recognize diverse gestures more accurately.
4. Learning effectiveness: feedback on the application’s efficacy in facilitating sign language learning was gathered from users. The chart in Figure 9.
5. Additional feedback: users were given the opportunity to provide supplementary comments, suggestions, and critiques. Suggestions primarily focus on enhancing user experience and functionality. Addressing latency issues and refining the user interface (UI) are paramount for improving overall satisfaction. Additionally, the implementation of offline access would accommodate users with limited internet connectivity. Enhancements in navigation, responsiveness, and clarity are also recommended to ensure an intuitive and efficient user experience.

**Figure 10:**
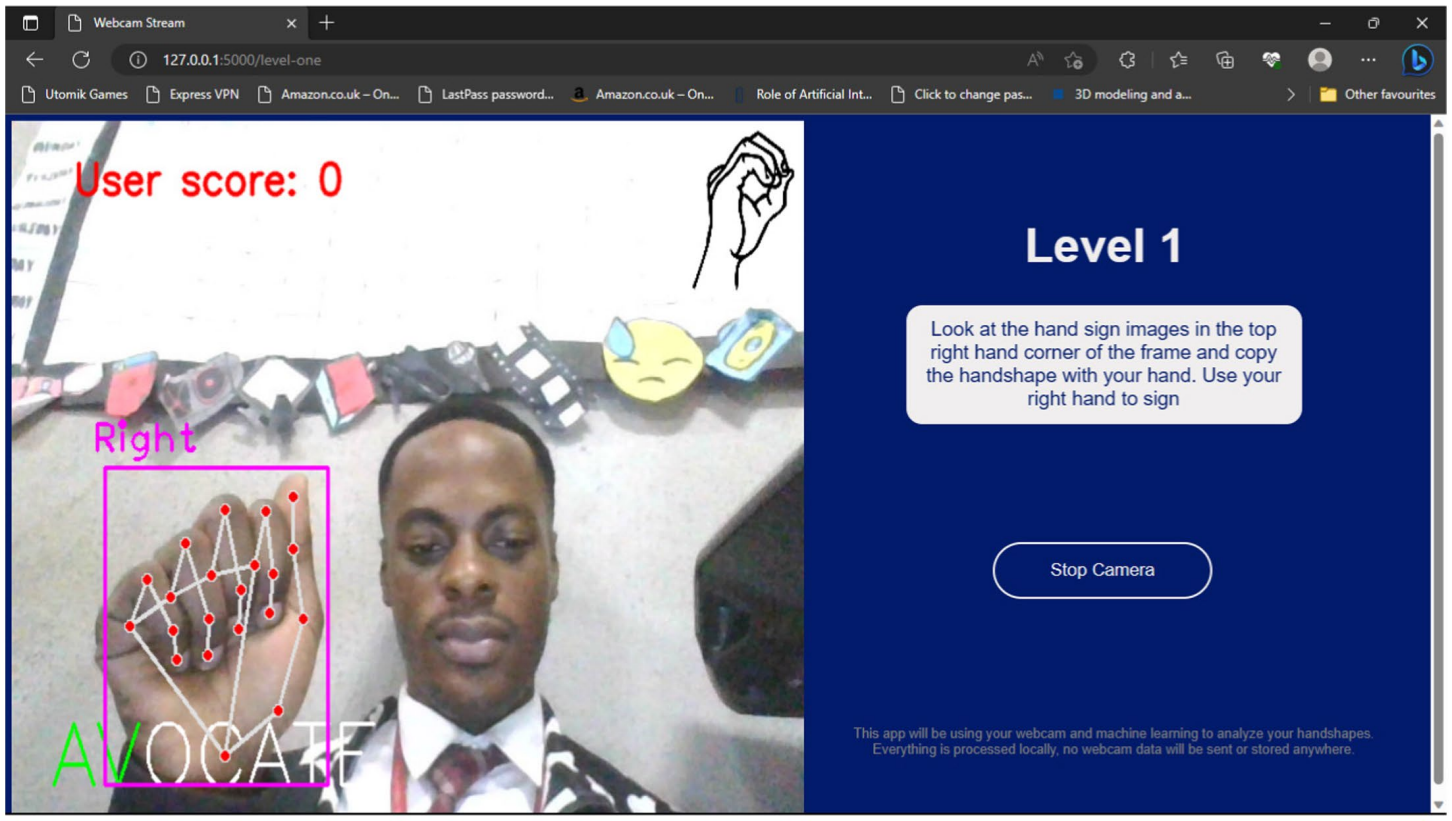
Level 1 page.

**Figure 11:**
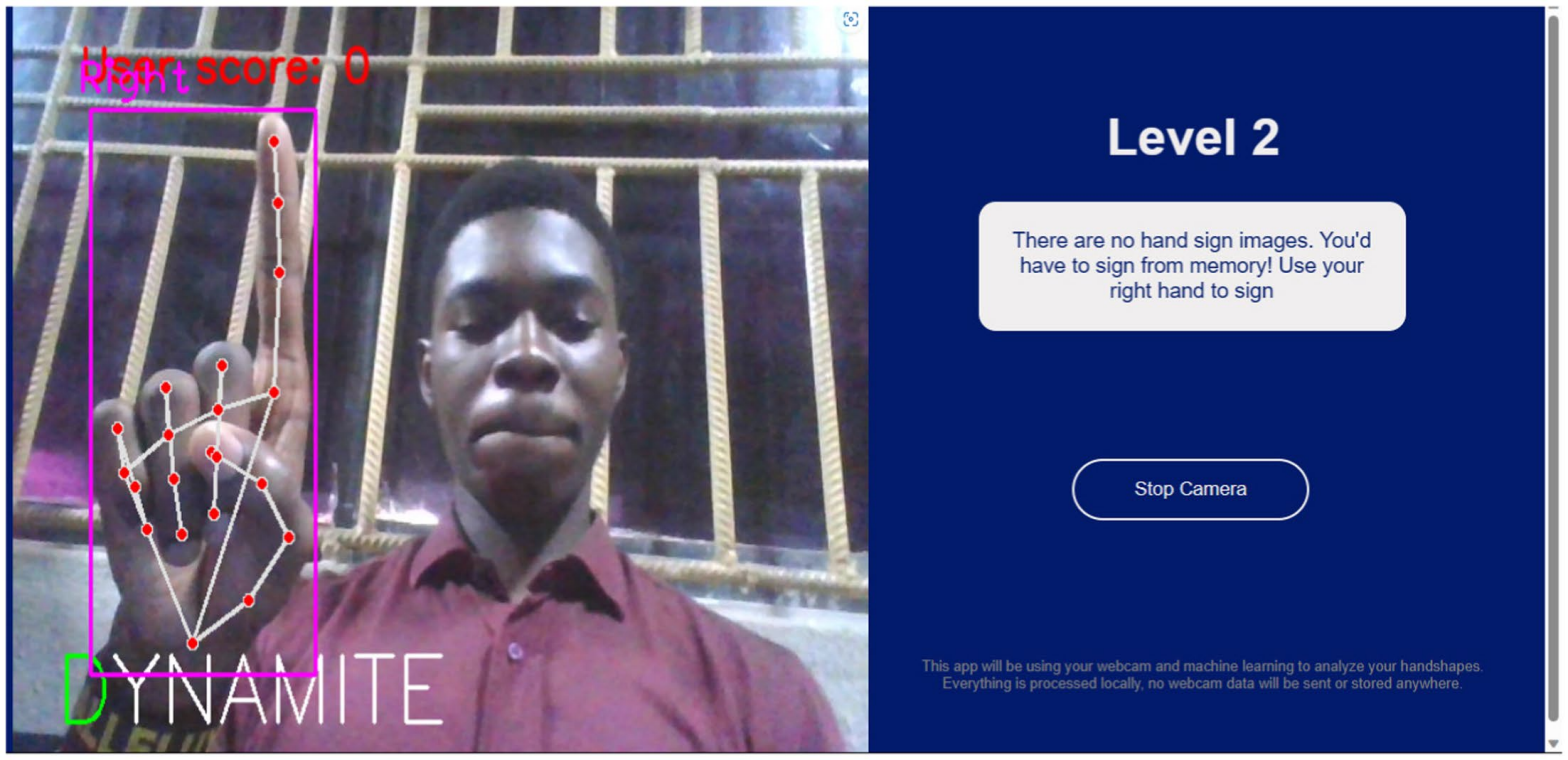
Level 2 page.

Figures 10, 11, respectively, show the Level 1 and Level 2 pages.

## 5 A comparative analysis

Table 3 shows a detailed analysis of the deployed system compared to previous works.

**Table 3:** Comparison between the deployed system and previous works.

Aspect	Your system	Previous works
Integration into web-based application	Successfully deployed as a web app	Primarily focused on offline or specialized settings
Accessibility and usability	Enhanced accessibility and usability	Limited exploration of web-based applications
User-friendly interface	Clear instructions, intuitive design	Varied levels of emphasis on user interface design
Scope of recognition	Focused on ASL alphabet gestures	Varied, with some studies addressing broader sign gestures
Average latency	Low latency, prompt response	Latency varies across studies, not explicitly discussed

## 6 Conclusion

The primary aim of this paper was to extend our previous work on sign language recognition by successfully deploying the application on the web. The web-based system represents a significant advancement in making sign language communication more accessible and user-friendly. We anticipate this work will contribute to the broader accessibility of sign language recognition technology and open new research and application development avenues in this field. The results and discussion section demonstrated the viability of the web-based application, showcasing comparable accuracy levels to previous studies. The low average latency observed in the system’s responsiveness further enhances its user-friendly experience. Visual elements, including screenshots of the application and a flowchart, provided a tangible representation of the research findings.

In charting a course for future endeavours, several avenues for improvement beckon. Firstly, the system’s scope could extend beyond the confines of the ASL alphabet, embracing a wider range of complex sign language gestures to enable more comprehensive communication. Beyond this, the integration of auxiliary assistive technologies, such as speech-to-text, presents an opportunity to create a more holistic communication solution. Additionally, the creation of a dedicated mobile or desktop application could further enhance accessibility, enabling users to engage with the system on the go. Also, by forging partnerships with organizations that cater to individuals with hearing disabilities, the system’s reach and impact can be significantly amplified, thereby fostering broader adoption.

## Data availability statement

The raw data supporting the conclusions of this article will be made available by the authors, without undue reservation.

## Ethics statement

Written informed consent was obtained from the individual(s) for the publication of any potentially identifiable images or data included in this article.

## Author contributions

HO: Supervision, Writing – review & editing. OI: Software, Writing – original draft, Methodology. JA: Software, Writing – original draft, Conceptualization, Visualization, Writing – review & editing.
